# The Impact of Baseline Risk Factors on the Incidence of Febrile Neutropenia in Breast Cancer Patients Receiving Chemotherapy with Pegfilgrastim Prophylaxis: A Real-World Data Analysis

**DOI:** 10.36469/001c.24564

**Published:** 2021-06-22

**Authors:** Edward Li, Bridgette Kanz Schroader, David Campbell, Kim Campbell, Weijia Wang

**Affiliations:** 1 Sandoz, Inc, Princeton, NJ, US; 2 Xcenda, LLC, Palm Harbor, FL, US

**Keywords:** real-world evidence, risk factors, febrile neutropenia, pegfilgrastim, granulocyte colony-stimulating factor

## Abstract

**Background:** There are sparse data addressing whether standard risk factors for febrile neutropenia (FN) are relevant in patients receiving myelosuppressive chemotherapy and primary prophylaxis for FN, which would have implications for variables to consider during real-world comparative analyses of FN incidence.

**Objective:** To assess the impact of baseline patient-specific risk factors and regimen risk on the incidence of FN in patients receiving pegfilgrastim primary prophylaxis.

**Methods:** This was a retrospective observational study in patients with breast cancer (BC) who received myelosuppressive chemotherapy and prophylactic pegfilgrastim identified January 1, 2017-May 31, 2018 from MarketScan® research databases. The outcomes were defined as incidence of FN in the first cycle and among all cycles of chemotherapy using three different definitions for FN. Logistic regression and generalized estimating equations models were used to compare outcomes among patients with and without patient-specific risk factors and among those receiving regimens categorized as high-, intermediate-, or other-risk for FN (low-risk or undefinable by clinical practice guidelines).

**Results:** A total of 4460 patients were identified. In the first cycle of therapy, patients receiving intermediate-risk regimens were at up to 2 times higher risk for FN across all definitions than those receiving high-risk regimens (*P*<0.01). The odds ratio for main FN among patients with ≥4 versus 0 risk factors was 15.8 (95% confidence interval [CI]: 1.5, 169.4; *P*<0.01). Patients with ≥3 FN risk factors had significantly greater risks for FN across all cycles of treatment than those with no risk factors; this was true for all FN definitions.

**Discussion:** The choice of FN definition significantly changed the impact of risk factors on the FN outcomes in our study, demonstrating the importance of evaluating all proxies for true FN events in a database study. This is particularly important during real-world study planning where potential missteps may lead to bias or confounding effects that render a study meaningless.

**Conclusions:** In patients with BC receiving chemotherapy with pegfilgrastim prophylaxis, patient-specific risk factors and regimen risk levels are determinants of FN risk. In real-world studies evaluating FN incidence, it is imperative to consider and control for these risk factors when conducting comparative analyses.

## INTRODUCTION

Febrile neutropenia (FN) is associated with increased morbidity and mortality in patients receiving myelosuppressive chemotherapy.^1–4^ Real-world evidence has reported an overall risk of mortality up to 11% after 1 or more hospitalizations for FN.^3^ The risk for, and severity of FN depends on the chemotherapy regimen, dose intensity, and patient-specific risk factors.^5^

Clinical practice guidelines published by the National Comprehensive Cancer Network (NCCN) stratify chemotherapy regimens into three FN risk categories as high risk (>20%), intermediate risk (10%–20%), and low risk (<10%) based on the agents, dose, and patient-specific risk factors. Employing these categories, they recommend the use of granulocyte colony-stimulating factors (G-CSFs), including short-acting (eg, filgrastim) and long-acting (eg, pegfilgrastim) formulations, as primary FN prophylaxis in all patients receiving high-risk regimens and for patients receiving intermediate-risk regimens who have at least one additional risk factor.^6^

An FN risk model by Lyman et al demonstrated strong association between risk factors and the incidence of FN.^7^ In the model, over 50% of patients classified at an intermediate risk level for FN based on regimen selection alone were subsequently re-classified as high risk when utilizing the risk model, which incorporated additional patient-, disease-, and treatment-related factors. Because of these results, clinical practice guidelines recommend consideration of the following risk factors when deciding upon the use of primary FN prophylaxis: age ≥65 years old, advanced disease, previous chemotherapy or radiation therapy, preexisting neutropenia or bone marrow involvement with tumor, infection, open wounds or recent surgery, poor performance or nutritional status, poor renal function, liver dysfunction, cardiovascular disease, human immunodeficiency virus (HIV) infection, and multiple comorbid conditions.^6,8,9^ While these patient-specific risk factors and the consequences of FN are universally acknowledged, implementation of risk factor analysis when deciding on primary prophylaxis with G-CSFs in individual patient cases remains subjective and inconsistent.^10–13^

Because of their influence on FN incidence, patient-specific risk factors and regimen risk level are imperative to consider as confounding variables in real-world comparative effectiveness analyses. With the introduction of six Food and Drug Administration (FDA)-approved biosimilar filgrastim and pegfilgrastim products, real-world data analyses will play an important role in provider confidence in treatment patterns or effectiveness in this multisource environment.^14–19^ Best practice dictates that prior to completing comparative effectiveness analyses, identifying and controlling for these potential confounding variables is essential to confidently report treatment differences in the incidence of FN.^20^

In order to understand how FN risk factors could potentially confound outcomes in real-world data analysis of myeloid growth factors, this study assessed the impact of baseline FN patient-specific risk factors and regimen risk levels on the incidence of FN in patients receiving pegfilgrastim as primary prophylaxis.

## METHODS

### Study Design and Data Source

This was a retrospective, observational, database study in patients with breast cancer (BC) who received myelosuppressive chemotherapy and primary prophylactic use of pegfilgrastim. The IBM MarketScan Commercial Claims and Encounters database and IBM MarketScan Medicare Supplemental database (IBM MarketScan research databases) were utilized. These de-identified national claims databases include over 150 large employers and health insurance plans containing more than 200 million subjects. In addition to the Medicare population, this dataset represents >15% of the employer-sponsored, and privately-insured United States (US) population who are aged <65 years old. Medical data, pharmacy data, and enrollment information were collected.^21^

### Patient and Cohort Identification

**Figure 1.**
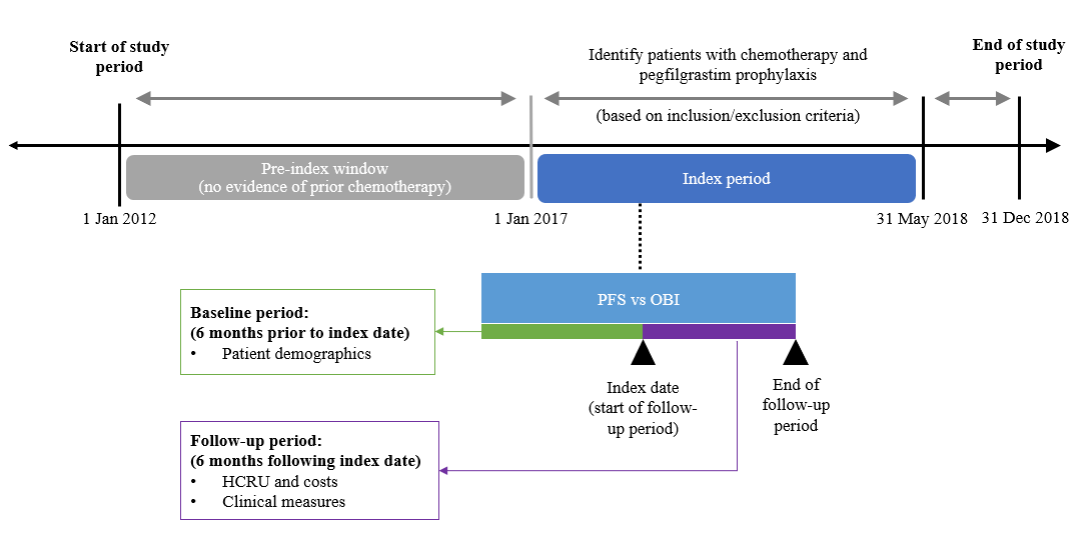
Study Design Schematic Abbreviations: HCRU, healthcare resource utilization; OBI, on-body injector; PFS, prefilled syringe.

The study period was from January 1, 2012 to December 31, 2018; adult commercial and Medicare health plan members who were diagnosed with BC and received chemotherapy with pegfilgrastim prophylaxis from January 1, 2017 to May 31, 2018 were identified. The first use of pegfilgrastim prophylaxis was defined as the index date; pegfilgrastim prophylaxis was defined as ≥1 claim for either medical or pharmacy pegfilgrastim ≤5 days after the chemotherapy cycle start date or cycle end date, whichever came first. Patients were required to be continuously enrolled at least 6 months prior to (baseline period) and following the index date (follow-up period). Additionally, patients had to be chemotherapy-free for 5 years prior to the first chemotherapy administration identified during the index period (Figure 1), which ensured that all patients included in this study exclusively received primary FN prophylaxis and not secondary prophylaxis.

Evidence of BC was defined as ≥1 diagnosis of cancer prior to the index date, via either inpatient or outpatient services. Chemotherapy was defined as ≥1 claim for a systemic cancer agent identified from medical outpatient services during the index period. The chemotherapy start date (cycle 1) was defined as the date of the first claim for a cancer treatment after the cancer diagnosis date but during the study period; the regimen was composed of all treatments filled or infused within the first 5 days after the start of the regimen; the end date was the earliest of the following: day before next medication administration (start of next cycle), start of a new regimen (end date considered the day prior), discontinuation of all agents in a regimen (runout date considered the last date of administration plus 29 days for infused drugs and the days’ supply minus 1 for filled drugs), end of study period, disenrollment from the health plan, or surgery or radiation (end date considered the day prior for both). Patients who received pegfilgrastim via the on-body injector (OBI) method of administration were assigned a day of pegfilgrastim administration 1 day later than the billing claim date due to the intended delivery of pegfilgrastim by the OBI device approximately 27 hours after application to the patient’s skin.

Patients were excluded if they had evidence of other malignancies; were male, pregnant, participating in a clinical trial, or HIV-positive; had a prior history of hematopoietic stem cell transplant; or had missing values of baseline and clinical characteristics.

### Outcome Measures

The main objective was to assess the impact of baseline patient-specific FN risk factors and chemotherapy regimen risk on the incidence of FN. Risk factors for FN were identified via medical claims in the baseline period and included metastatic disease to the bone, baseline radiation, recent surgery, baseline liver dysfunction, baseline renal dysfunction, history of persistent neutropenia, and age ≥65 during the index year. Chemotherapy regimen risk levels for FN were categorized as high, intermediate, and other (including low and undefinable) utilizing the NCCN guidelines. Regimens and their associated FN risk utilized in this study are listed in the **Supplemental Appendix**. Of note, patients who received dose-dense regimens were identified by the time period between cycles (14-day regimens were considered dose-dense).

FN events were captured in each chemotherapy cycle from both medical inpatient and outpatient services by proxy using diagnosis codes for neutropenia, fever, and/or infection, as previously validated by Weycker et al.^22^ Since FN outcomes can differ by the definition of FN that is chosen, three common definitions of FN were used based on past database studies: (1.) main definition: diagnosis code for neutropenia in position 1 or 2; (2.) sensitive definition: diagnosis code for neutropenia in position 1 or 2, with fever or infection on the same claim; (3.) specific definition: diagnosis code for neutropenia in position 1 or 2 with fever on the same claim. The use of these definitions to identify patients with FN from administrative claims data has been verified previously and is the standard approach.^22,23^ FN incidence was calculated based on the number of FN events for the first chemotherapy cycle and among all chemotherapy cycles.

### Statistical Analysis

Descriptive statistics were provided for all study variables. To compare FN incidence among the first chemotherapy cycle, logistic regression models were developed, adjusting for geographic region, chemotherapy regimen FN risk category (high, intermediate, other), Quan-Charlson comorbidity scores, days between chemotherapy and pegfilgrastim administration, and the number of patient-specific risk factors.

To compare the FN incidence among all chemotherapy cycles, generalized estimating equations (GEE) models with binomial distribution, log-link function, and exchangeable correlation structure were developed to account for the fact that the probability of experiencing an FN event during one chemotherapy cycle was dependent on previous chemotherapy cycles (ie, the assumption of independent samples was violated). The GEE models estimated the average FN incidence across all chemotherapy cycles and controlled for the same covariates as the logistic regression model.

## RESULTS

### Patient Characteristics

A total of 4460 patients with BC were identified (Table 1). The median age was 54 years (range [minimum, maximum]: 23–69), and the majority of patients resided in the South region (50.5%). The population consisted of mainly commercially insured patients (93.2%) receiving high-risk chemotherapy regimens (83.6%). The mean number of days per cycle per patient in cycle one was 19.9 days (standard deviation [SD]: 22.4); the mean number of total days in all cycles per patient was 78.1 days (SD: 62.6). The mean number of patients per cycle ranged from 4460 in the first cycle to 3329 in the third cycle and 619 in the sixth cycle (final cycle). Baseline characteristics of the overall population, those who experienced FN in the first chemotherapy cycle, and those who experienced FN in any chemotherapy cycle are shown in Table 2. FN incidence by number of risk factors and level of chemotherapy regimen risk are shown in Table 3.

**Table 1.** Sample Attrition Study Diagram

**Unique Patients**		
**Chemotherapy**	Medical outpatient 1/1/2017–5/31/2018	218 716
	No chemotherapy 5 years prior	122 112
**Pegfilgrastim**	Pegfilgrastim 1/1/2017–5/31/2018 (index date)	26 283
**Join**	Chemotherapy and pegfilgrastim ≤5 days apart	12 880
**Enrollment**	6 months prior to index date	9854
	6 months following index date	8001
**Adult**	Adult on index year	7968
**Cancer**	≥1 diagnosis of cancer prior to index date	5667
**Exclusion**	Myeloid cancer	5642
	Pregnancy	5539
	Clinical trial participation	5427
	HIV positive	5408
	Hematopoietic cell transplantation	5385
	Other types of non-myeloid cancers	4849
	Missing region	4840
**Final total**	4460	

Abbreviations: HIV, human immunodeficiency virus.

**Table 2.** Baseline Characteristics of Female Breast Cancer Patients and Patients with FN

**Variable**	**Count, n (%)**	**Patients with FN: First Chemotherapy Cycle, n (%)**			**Patients with FN: All Chemotherapy Cycles, n (%)**		
		**Main Definition**	**Sensitive Definition**	**Specific Definition**	**Main Definition**	**Sensitive Definition**	**Specific Definition**
**Total Population**	**4460 (100)**	**183 (4.10)**	**61 (1.37)**	**57 (1.28)**	**574 (3.65)**	**163 (1.04)**	**149 (0.95)**
**US Region**							
North Central	921 (20.65)	38 (4.13)	13 (1.41)	13 (1.41)	129 (4.01)	35 (1.09)	32 (1.00)
Northeast	721 (16.17)	39 (5.41)	11 (1.53)	10 (1.39)	121 (4.98)	32 (1.32)	27 (1.11)
South	2252 (50.49)	83 (3.69)	30 (1.33)	29 (1.29)	253 (3.15)	68 (0.85)	67 (0.83)
West	566 (12.69)	23 (4.06)	7 (1.24)	NR^a^	71 (3.46)	28 (1.36)	23 (1.12)
**Insurance Type**							
Commercial	4157 (93.21)	168 (4.04)	53 (1.27)	49 (1.18)	535 (3.65)	148 (1.01)	135 (0.92)
Medicare	303 (6.79)	15 (4.95)	8 (2.64)	8 (2.64)	39 (3.73)	15 (1.43)	14 (1.34)
**Regimen Risk Leve**l							
High	3727 (83.57)	139 (3.73)	44 (1.18)	41 (1.10)	491 (3.48)	136 (0.96)	124 (0.88)
Intermediate	667 (14.96)	41 (6.15)	16 (2.40)	15 (2.25)	75 (5.40)	24 (1.73)	22 (1.58)
Other^b^	66 (1.48)	NR^a^	NR^a^	NR^a^	8 (3.96)	NR^a^	NR^a^
**Quan-Charlson Comorbidity Score**							
1–2	1687 (35.09)	62 (3.98)	17 (1.09)	16 (1.03)	199 (3.49)	46 (0.81)	44 (0.77)
3–4	800 (16.64)	28 (4.17)	9 (1.34)	7 (1.04)	84 (3.66)	21 (0.92)	16 (0.70)
5+	2321 (48.27)	93 (4.17)	35 (1.57)	34 (1.52)	291 (3.76)	96 (1.24)	89 (1.15)
**Days between Chemotherapy and Pegfilgrastim Administration**							
D0	1974 (44.26)	92 (4.66)	25 (1.27)	25 (1.27)	278 (4.08)	63 (0.92)	56 (0.82)
D1	2340 (52.47)	85 (3.63)	33 (1.41)	31 (1.32)	279 (3.29)	96 (1.13)	91 (1.07)
D2+	146 (3.27)	6 (4.11)	NR^a^	NR^a^	17 (4.02)	NR^a^	NR^a^
**Metastatic Disease to the Bone**							
No	4459 (99.98)	183 (4.10)	61 (1.37)	57 (1.28)	574 (3.65)	163 (1.04)	149 (0.95)
Yes	NR^a^	0 (0.00)	0 (0.00)	0 (0.00)	0 (0.00)	0 (0.00)	0 (0.00)
**Baseline Radiation**							
No	4313 (96.70)	177 (4.10)	59 (1.37)	55 (1.28)	546 (3.59)	156 (1.03)	142 (0.93)
Yes	147 (3.30)	NR^a^	NR^a^	NR^a^	28 (5.43)	7 (1.36)	7 (1.36)
**Recent Surgery**							
No	714 (16.01)	31 (4.34)	9 (1.26)	8 (1.12)	88 (3.78)	19 (0.82)	18 (0.77)
Yes	3746 (83.99)	152 (4.06)	52 (1.39)	49 (1.31)	486 (3.63)	144 (1.08)	131 (0.98)
**Baseline Liver Dysfunction**							
No	4139 (92.80)	162 (3.91)	55 (1.33)	52 (1.26)	509 (3.49)	152 (1.04)	141 (0.97)
Yes	321 (7.20)	21 (6.54)	NR^a^	NR^a^	65 (5.77)	11 (0.98)	8 (0.71)
**Baseline Renal Dysfunction**							
No	4344 (97.40)	177 (4.07)	59 (1.36)	52 (1.26)	551 (3.60)	154 (1.01)	141 (0.92)
Yes	116 (2.60)	NR^a^	NR^a^	NR^a^	23 (5.45)	9 (2.13)	8 (1.90)
**History of Persistent Neutropenia**							
No	4440 (99.55)	165 (3.72)	60 (1.35)	56 (1.26)	518 (3.31)	162 (1.04)	148 (0.95)
Yes	20 (0.45)	18 (90.00)	NR^a^	NR^a^	56 (76.71)	NR^a^	NR^a^
**Age ≥65 Years Old**							
No	4114 (92.24)	167 (4.06)	52 (1.26)	48 (1.17)	530 (3.65)	145 (1.00)	132 (0.91)
Yes	346 (7.76)	16 (4.62)	9 (2.60)	9 (2.60)	44 (3.66)	18 (1.50)	17 (1.41)

Abbreviations: FN, febrile neutropenia; NR, not reported; US, United States.

^a^ NR: Results with n values ≥1 to ≤6 are not reported to maintain patient anonymity.

^b^ Other risk includes low risk of FN according to National Comprehensive Cancer Network guideline and regimens whose risk of FN cannot be defined.

**Table 3.** FN Incidence by Number of Risk Factors and Level of Regimen Risk

**Variable**	**Total Population, n (%)**	**Patients with FN: First Chemotherapy Cycle**			**Patients with FN: All Chemotherapy Cycles**		
		**Main Definition**	**Sensitive Definition**	**Specific Definition**	**Main Definition**	**Sensitive Definition**	**Specific Definition**
**Number of Risk Factors**							
0	529 (11.79)	18 (3.43)	NR^a^	NR^a^	55 (3.29)	13 (0.78)	13 (0.78)
1	3255 (72.56)	121 (3.73)	46 (1.42)	42 (1.30)	356 (3.06)	116 (1.00)	106 (0.91)
2	639 (14.24)	35 (5.52)	7 (1.10)	7 (1.10)	145 (6.62)	28 (1.28)	24 (1.10)
3	57 (1.27)	8 (14.29)	NR^a^	NR^a^	16 (7.55)	NR^a^	NR^a^
4	5 (0.11)	NR^a^	0 (0)	0 (0)	NR^a^	0 (0)	0 (0)
5	NR^a^	0 (0)	0 (0)	0 (0)	0 (0)	0 (0)	0 (0)
1+	3957 (88.21)	165 (4.19)	57 (1.45)	53 (1.35)	519 (3.69)	150 (1.07)	136 (0.97)
2+	702 (15.65)	44 (6.33)	11 (1.58)	11 (1.58)	163 (6.73)	34 (1.40)	30 (1.24)
3+	63 (1.40)	9 (14.75)	NR^a^	NR^a^	18 (7.69)	NR^a^	NR^a^
4+	NR^a^	NR^a^	0 (0)	0 (0)	NR^a^	0 (0)	0 (0)
**Regimen Risk: Other**							
0	14 (0.31)	0 (0)	0 (0)	0 (0)	0 (0)	0 (0)	0 (0)
1	41 (0.91)	NR^a^	0 (0)	0 (0)	7 (5.88)	NR^a^	NR^a^
2	8 (0.18)	0 (0)	0 (0)	0 (0)	0 (0)	0 (0)	0 (0)
3	NR^a^	NR^a^	NR^a^	NR^a^	NR^a^	NR^a^	NR^a^
4	0 (0)	0 (0)	0 (0)	0 (0)	0 (0)	0 (0)	0 (0)
5	0 (0)	0 (0)	0 (0)	0 (0)	0 (0)	0 (0)	0 (0)
1+	52 (1.16)	NR^a^	NR^a^	NR^a^	8 (5.00)	NR^a^	NR^a^
2+	11 (0.25)	NR^a^	NR^a^	NR^a^	NR^a^	NR^a^	NR^a^
3+	NR^a^	NR^a^	NR^a^	NR^a^	NR^a^	NR^a^	NR^a^
4+	0 (0)	0 (0)	0 (0)	0 (0)	0 (0)	0 (0)	0 (0)
**Regimen Risk: Intermediate**							
0	67 (1.49)	NR^a^	NR^a^	NR^a^	NR^a^	NR^a^	NR^a^
1	488 (10.88)	33 (6.76)	14 (2.87)	13 (2.66)	54 (5.43)	17 (1.71)	16 (1.61)
2	104 (2.32)	NR^a^	NR^a^	NR^a^	14 (5.60)	NR^a^	NR^a^
3	7 (0.16)	NR^a^	0 (0)	0 (0)	NR^a^	NR^a^	NR^a^
4	NR^a^	0 (0)	0 (0)	0 (0)	0 (0)	0 (0)	0 (0)
5	NR^a^	0 (0)	0 (0)	0 (0)	0 (0)	0 (0)	0 (0)
1+	601 (13.40)	39 (6.50)	15 (2.50)	14 (2.33)	70 (5.49)	22 (1.73)	20 (1.57)
2+	113 (2.52)	NR^a^	NR^a^	NR^a^	16 (5.71)	NR^a^	NR^a^
3+	9 (0.20)	NR^a^	0 (0)	0 (0)	NR^a^	NR^a^	NR^a^
4+	NR^a^	0 (0)	0 (0)	0 (0)	0 (0)	0 (0)	0 (0)
**Regimen Risk: High**							
0	448 (9.99)	16 (3.60)	NR^a^	NR^a^	50 (3.31)	11 (0.73)	11 (0.73)
1	2726 (60.77)	86 (3.17)	32 (1.18)	29 (1.07)	295 (2.81)	97 (0.92)	88 (0.84)
2	527 (11.75)	30 (5.75)	NR^a^	NR^a^	131 (6.88)	24 (1.26)	21 (1.10)
3	47 (1.05)	NR^a^	NR^a^	NR^a^	13 (7.18)	NR^a^	NR^a^
4	NR^a^	NR^a^	0 (0)	0 (0)	NR^a^	0 (0)	0 (0)
5	0 (0)	0 (0)	0 (0)	0 (0)	0 (0)	0 (0)	0 (0)
1+	3304 (73.65)	123 (3.75)	41 (1.25)	38 (1.16)	441 (3.50)	125 (0.99)	113 (0.90)
2+	578 (12.88)	37 (6.47)	9 (1.57)	9 (1.57)	146 (6.95)	28 (1.33)	25 (1.19)
3+	51 (1.14)	7 (14.00)	NR^a^	NR^a^	15 (7.61	NR^a^	NR^a^
4+	NR^a^	NR^a^	0 (0)	0 (0)	NR^a^	0 (0)	0 (0)

Abbreviations: FN, febrile neutropenia; NR, not reported.

^a^ NR: Results with n values ≥1 to ≤6 are not reported to maintain patient anonymity.

### First Cycle FN Incidence: Logistic Regression Adjusted Odds Ratio

The results of the logistic regressions showed that patients receiving intermediate-risk regimens had a significantly higher risk for first cycle FN across all FN definitions. The odds ratios (ORs) of first cycle FN for patients receiving intermediate- versus high-risk chemotherapy regimens ranged from 1.7 (95% confidence interval [CI]: 1.2, 2.5) with the main FN definition, to 2.1 (95% CI: 1.1, 3.7) with the sensitive FN definition (all *P*<0.01).

The days between chemotherapy and pegfilgrastim administration (day 0 vs day 1) were not significantly associated with risk of FN using any definition. Patients in the Northeast versus South region had a significantly greater FN incidence using the main definition only (Table 3).

Patients with increasing numbers of risk factors for FN had significantly higher probabilities of FN compared to those with no risk factors across all FN definitions (Table 4). The ORs for the main definition ranged from 3.9 (1.7–8.7) among those with >=1 versus 0 risk factors to 15.8 (95% CI: 1.5, 169.4) among those with >=4 versus 0 risk factors (all *P*<0.01). The ORs when comparing >=3 versus 0 risk factors were 6.1 (95% CI: 1.2, 29.9) for the specific definition, 6.4 (95% CI: 1.3, 31.2) for the sensitive definition, and 10.1 (95% CI: 2.6, 39.3) for the main definition. This pattern was consistent when comparing patients with >=3 versus 2 risk factors across all FN definitions.

**Table 4.** Logistic Regression Adjusted Odds Ratio of FN Incidence Among the First Cycle

**Variable**	**Main Definition OR (95% CI)**			**Sensitive Definition OR (95% CI)**			**Specific Definition OR (95% CI)**		
Region: Northeast vs South	1.57^a^ (1.05, 2.33)			1.12 (0.55, 2.28)			1.06 (0.51, 2.22)		
Regimen Risk: Intermediate vs High	1.72^a^ (1.20, 2.48)			2.05^a^ (1.14, 3.68)			2.00^a^ (1.09, 3.66)		
Days between Chemo and Peg Admin: D0 vs D1	1.29 (0.95, 1.75)			0.87 (0.51, 1.48)			0.92 (0.54, 1.57)		
	**Main Definition, N=4459^b^ OR (95% CI)**			**Sensitive Definition, N=4455^c^ OR (95% CI)**			**Specific Definition, N=4455^c^ OR (95% CI)**		
**Number of Risk Factors**	**0**	**1**	**2**	**0**	**1**	**2**	**0**	**1**	**2**
≥4	15.77^a^ (1.47, 169.39)	13.85^a^ (1.35, 142.02)	8.45 (0.83, 86.04)	--	--	--	--	--	--
≥3	10.10^a^ (2.59, 39.32)	8.87^a^ (2.47, 31.88)	5.41^a^ (1.51, 19.34)	6.35^a^ (1.29, 31.17)	3.46 (0.99, 12.17)	5.76a (1.56, 21.25)	6.09^a^ (1.24, 29.86)	3.80^a^ (1.08, 13.39)	5.76^a^ (1.56, 21.18)
≥2	5.76^a^ (2.12, 15.63)	5.06^a^ (2.08, 12.31)	--	2.65 (0.72, 9.72)	1.44 (0.61, 3.44)	--	2.54 (0.69, 9.36)	1.58 (0.66, 3.81)	--
≥1	3.84^a^ (1.70, 8.66)	--	--	2.34 (0.74, 7.38)	--	--	2.18 (0.69, 6.88)	--	--

Abbreviations: Chemo, chemotherapy; CI, confidence interval; FN, febrile neutropenia; OR, odds ratio; Peg, pegfilgrastim.

^a^ Statistically significant.

^b^ Baseline renal dysfunction was omitted from this model; this restriction was applied to avoid the model failing to converge.

^c^ Baseline liver and renal dysfunction were omitted from these models; these restrictions were applied to avoid the models failing to converge.

### All Cycles FN Incidence: GEE Model Adjusted OR

Across patient cohorts, patients who received intermediate-risk regimens had greater FN rates across all cycles compared to patients treated with high-risk regimens. The ORs ranged from 1.6 (95% CI: 1.1, 2.2) with the main definition to 1.8 (95% CI: 1.1, 2.8) with the sensitive definition (all *P*<0.05). Patients residing in the Northeast and North central regions versus South region had a higher incidence of FN across all chemotherapy cycles, although not statistically different. Patients with ≥3 FN risk factors had a greater risk for FN across all cycles than those with no risk factors; the ORs were 3.3 (95% CI: 1.2, 9.0) for the specific definition, 3.3 (95% CI: 1.2, 8.9) for the sensitive definition, and 3.4 (95% CI: 1.0, 12.2) for the main definition, *P*<0.05 for both the specific and sensitive definitions (Table 5). There was a consistent pattern, although not as strong as seen in the first-cycle analysis, that as the number of patient-specific risk factors increased, the odds of experiencing FN also increased among all chemotherapy cycles.

**Table 5.** GEE Adjusted Odds Ratio of FN Incidence Among All Cycles

**Variable**	**Main Definition OR (95% CI)**			**Sensitive Definition OR (95% CI)**			**Specific Definition OR (95% CI)**		
Region: North- east vs South	1.63^a^ (1.15, 2.32)			1.57 (0.97, 2.54)			1.34 (0.83, 2.17)		
Region: North Central vs South	1.30 (0.94, 1.82)			1.27 (0.82, 1.96)			1.16 (0.74, 1.83)		
Regimen Risk: Intermediate vs High	1.58^a^ (1.11, 2.24)			1.75^a^ (1.09, 2.79)			1.74^a^ (1.07, 2.85)		
Days between Chemo and Peg Admin: D0 vs D1	1.23 (0.96, 1.58)			0.82 (0.58, 1.16)			0.76 (0.54, 1.09)		
	**Main Definition, Cycles=15712^b^ OR (95% CI)**			**Sensitive Definition, Cycles=15696^c^ OR (95% CI)**			**Specific Definition, Cycles=15696^c^ OR (95% CI)**		
**Number of risk factors**	**0**	**1**	**2**	**0**	**1**	**2**	**0**	**1**	**2**
≥4	4.70 (0.46, 48.07)	4.91 (0.50, 48.74)	2.25 (0.22, 22.51)	--	--	--	--	--	--
≥3	3.42 (0.96, 12.19)	3.57^a^ (1.06, 12.06)	1.63 (0.48, 5.61)	3.25^a^ (1.19, 8.85)	2.47^a^ (1.07, 5.69)	2.04 (0.82, 5.06)	3.28^a^ (1.20, 8.96)	2.78^a^ (1.20, 6.42)	2.38 (0.95, 5.94)
≥2	2.90^a^ (1.17, 7.20)	3.04^a^ (1.33, 6.95)	--	2.27^a^ (1.07, 4.81)	1.73^a^ (1.05, 2.85)	--	2.13^a^ (1.00, 4.51)	1.80^a^ (1.08, 3.00)	--
≥1	2.20^a^ (1.06, 4.57)	--	--	1.90 (0.97, 3.70)	--	--	1.75 (0.90, 3.42)	--	--

Abbreviations: Chemo, chemotherapy; CI, confidence interval; FN, febrile neutropenia; GEE, generalized estimating equation; OR , odds ratio; Peg, pegfilgrastim.

^a^ Statistically significant

^b^ Baseline renal dysfunction was omitted from this model; this restriction was applied to avoid the model failing to converge.

^c^ Baseline liver and renal dysfunction were omitted from this model; these restrictions were applied to avoid the model failing to converge.

## DISCUSSION

Overall, our findings confirm that chemotherapy regimen risk levels and patient-specific risk factors affect the incidence of FN in a population of BC patients undergoing myelosuppressive chemotherapy and receiving pegfilgrastim primary prophylaxis. In the case of those with 2 or more risk factors, patients were over 5 times more likely to experience first cycle FN than those without any risk factors.

The choice of FN definition significantly changed the impact of observed patient-specific risk factors on the FN outcomes in our study. For example, BC patients residing in the Northeast versus South region were significantly more likely to experience FN when using the main definition. However, this definition only considers a claim for neutropenia. Therefore, this difference could be representative of different practice patterns for assessing blood counts in these regions driven by differences in health-care coverage, adherence to treatment plans by patients, or racial or cultural differences and may not represent a true difference in our outcome of interest. Follow-up studies are required to evaluate these interesting differences in a well-designed, quasi-experimental fashion that controls for confounding variables to allow for causal inference. Nevertheless, it demonstrates how important it is to use all possible proxies for true FN events in a database study.

As the application of real-world evidence to inform decision-making increases, the importance of statistical pre-planning and appropriate use of real-world evidence does too. Christian et al proposed a framework based on the transferability of key randomized controlled trial design elements to real-world studies. Notably, the authors cited the importance of selecting data sources that are “fit for purpose” and the importance of researchers having familiarity with those datasets to identify potential sources of bias or misclassification. They also noted analytical methods should be transparent, preplanned, and optimized.^24^ Similarly, the FDA has issued guidance defining reliability as “whether sufficient data elements are collected to adjust for confounding factors that may impact the exposure or outcomes of interest,” clearly indicating any study without proper statistical controlling of bias and confounding effects would not be considered reliable by the FDA.^25^ These concepts are aligned with multiple regulatory-supported initiatives from the FDA and the European Medicines Agency, showing the growing interest in the use of real-world evidence and the need for robust analyses.^25–28^

These requirements for real-world analyses and the importance of experienced and informed researchers are akin to patients seeking clinicians and treatment centers with expertise in one disease area. In our study, if the difficulty of accurately identifying FN events in a claims-based study was not recognized, false or misleading conclusions could be drawn by only utilizing or relying on the basic main definition for FN events. These potential missteps are important to recognize during real-world study planning, particularly when the data will be utilized for comparative effectiveness analyses. A real-world study that purportedly demonstrates differences between two myeloid growth factors that utilizes certain FN definitions not commonly used or universally recognized would introduce severe bias or confounding effects that render the study meaningless.

For example, a real-world analysis by Gawade et al evaluated patterns of G-CSF prophylaxis in cancer patients receiving myelosuppressive chemotherapy. They concluded that patients who persistently received G-CSF throughout their chemotherapy courses had a lower risk of developing FN than those who discontinued early and that persistence may be more likely with the OBI compared to pre-filled syringe method of administration.^29^ However, the study design did not control for potential biases including confounding variables, changes to regimen dose intensity, or patient-specific risk factors. Reporting of these or similar unfounded results could then create major misinformation campaigns among payers and providers.

Next, when considering the impact of a chemotherapy regimen’s risk level on FN incidence, our results demonstrated that in a cohort of patients who received pegfilgrastim primary prophylaxis, those receiving intermediate-risk chemotherapy regimens were more likely to experience FN than those receiving high-risk regimens. Our study was not designed to test the causal inference between chemotherapy regimen risk and FN outcomes, and thus is a hypothesis-generating result that may be driven by a few reasons. First, the sample size of patients receiving intermediate regimens was small compared to those receiving high-risk regimens (15% vs 84% of the included patients, respectively) and the results may be driven by this small sample where bias would have a larger impact. Second, there may be factors that are unobserved in claims data that contribute to the observed difference but would be unaccounted for in our model. For example, providers may empirically dose reduce patients receiving high-risk regimens, decreasing their risk of FN. Last, we analyzed patient-specific risk factors as categorical variables, when in reality, they are more likely to be continuous variables. For example, we reported if patients had prior radiation, when in fact, the amount of prior radiation may also influence the risk of FN.

The potential impact of these results is still important to further assess as this notion is imperative for clinicians in practice. Clinical practice guidelines traditionally recommended primary prophylaxis for patients receiving intermediate-risk regimens if they have at least one risk factor for FN.^6^ Therefore, patients in this study, where an inclusion criteria was the receipt of primary prophylaxis, were already identified as having an increased risk of experiencing FN. The fact that this regimen risk factor remained significant despite the use of primary prophylaxis highlights the need for further research to deduce if close follow-up of these patients is warranted to help prevent the morbidity, and sometimes even mortality, associated with FN.

In our study, patients with increasing numbers of risk factors were significantly more likely to experience FN across all definitions in the first cycle and among all cycles of chemotherapy. This is aligned with previously published literature regarding FN incidence in patients receiving myelosuppressive chemotherapy.^12,30,31^ Averin et al conducted a retrospective observational study of 4091 patients receiving chemotherapy at four US health systems from 2009 to 2017.^30^ The majority of patients had at least one patient-specific FN risk factor (92%) and received high- or intermediate-risk regimens (20.5% and 30.8%, respectively). Their results showed that the incidence of FN is elevated in patients for whom primary prophylaxis is recommended but not received (16.9% in patients receiving high-risk regimens and 15.9% in patients receiving intermediate-risk regimens with at least one risk factor measured over all chemotherapy courses) and that FN incidence is frequently associated with severe and costly consequences such as hospitalization, which occurred in greater than 90% of cases in their study. Weycker et al sought to estimate the prevalence of risk factors for FN in patients receiving non-high-risk chemotherapy regimens through a retrospective analysis of two major US health-care claims repositories that included 160 304 patients receiving myelosuppressive chemotherapy (with or without primary FN prophylaxis) from 2003 to 2012.^31^ They concluded that most patients (74% to 98%) had at least one risk factor for FN and that, similar to this study, the risk of FN was increased among patients with risk factors, particularly those with multiple risk factors, compared to those without (relative risk range: 1.1 [95% CI: 0.8, 1.3], 2.2 [95% CI: 1.5, 3.0] [depending on oncologic indication]).

As with other real-world discussions, the economic impact of therapies must be considered due to the resource-restricted US health-care landscape. Recently, the Centers for Medicare & Medicaid Services requested information to further advance coordinated, high-quality, value-based care through the Oncology Care First model.^32^ Among the proposed changes, the Oncology Care First model would capitate payments, which appears to be an intermediate step on the journey toward bundled payments. Previous literature has supported the expanded use of biosimilars to promote value-based oncology care as means to benefit payers, providers, and patients.^33^ An analysis of biosimilar filgrastim for primary prophylaxis in patients receiving intermediate-risk, curative chemotherapy regimens for BC, non-small cell lung cancer, and non-Hodgkin lymphoma found it was cost-effective compared to secondary prophylaxis.^34^ Biosimilar access will be important to the health-care system, as the full benefits and savings biosimilars may provide will only be realized with continued growth in uptake and utilization. The economic implications of pegfilgrastim and biosimilar administration should be investigated in future health economics and outcomes research evaluating biosimilar versus reference products and including a cost component.

Importantly, the previously discussed studies and the majority of published literature regarding patient-specific FN risk factors were developed by studying patients who were not receiving primary prophylaxis.^30,31,35^ This study is the first, to our knowledge, to demonstrate the substantial impact of patient-specific risk factors on FN incidence in a population of patients all receiving pegfilgrastim primary prophylaxis in the United States. Because of this, it further validates the importance and absolute necessity of using statistical techniques (eg, propensity score matching) to control for these variables in any real-world study that purports to demonstrate a difference in FN incidence between two pegfilgrastim products. Failure to do so will lead to biased and untrue results, subsequently creating challenges for appropriate clinical practice decision-making.

### Limitations

Our study has several limitations to note. First, there are inherent limitations in database studies including misclassifications resulting in inaccurate or missing data. We excluded patients with missing clinical or baseline data, rigorously reviewed identified patients and chemotherapy regimens, and included all baseline variables in our statistical models to try to reduce confounding effects. Additionally, there is a risk of selection bias for patients receiving certain regimens, such as dose-dense chemotherapy. Patients selected for these regimens may be younger, fitter, and more likely to receive aggressive dosing; we attempted to control for this bias by including these baseline factors in our models.

Next, it is difficult to accurately identify patients experiencing FN. We utilized previously validated definitions of FN, but this highlights the importance of recognizing and acknowledging shortcomings in database studies to ensure that the data presented are an accurate representation of practice as previously discussed. This study is descriptive in nature, and our use of multiple definitions of FN provides more information for future researchers using claims data to consider when designing their study.

Because clinical practice guidelines do not routinely recommend primary pegfilgrastim prophylaxis in patients receiving intermediate-risk regimens without FN risk factors, our results are only valid for patients receiving primary prophylaxis with intermediate-risk regimens and are not externally valid for all patients receiving intermediate-risk regimens. Recent updates to clinical practice guidelines due to COVID-19 recommend primary prophylaxis with intermediate-risk regimens to reduce exposures to health-care settings; updated analyses using a dataset during the COVID-19 pandemic may yield more valid results for all patients receiving intermediate-risk regimens.^36,37^

Finally, we evaluated the effects of FN risk factors primarily identified by the Lyman model and recommended by NCCN for use in clinical practice. However, there may be additional significant risk factors unacknowledged in our model or confounders influencing the presence of risk factors that may be missing from this model.

## CONCLUSIONS

For patients with BC receiving chemotherapy with pegfilgrastim prophylaxis, geographic region, chemotherapy regimen risk of FN and the number of risk factors may affect FN incidence. For real-world studies attempting to compare the impact of pegfilgrastim products on FN incidence, this study demonstrates that it is critical to properly control for variables such as patient-specific risk factors and regimen-specific risk to estimate real differences in FN incidence among cohorts.

## Supplementary Material

Supplemental Appendix
