# Supplementary material for: The Impact of Baseline Risk Factors on the Incidence of Febrile Neutropenia in Breast Cancer Patients Receiving Chemotherapy with Pegfilgrastim Prophylaxis: A Real-World Data Analysis
Source: J Health Econ Outcomes Res. 2021 Jun 22;8(1):106–15. doi: 10.36469/001c.24564 (PMC8787317; doi:10.36469/001c.24564)
Supplement: Supplemental Appendix [file jheor_2021_8_1_24564_72977.pdf]

Journal of Health Economics and Outcomes Research

Supplementary Appendix

Li E, Schroader BK, Campbell D, Campbell K, Wang W. The impact of baseline risk factors on the incidence of febrile neutropenia in breast cancer patients receiving chemotherapy with pegfilgrastim prophylaxis: a real-world data analysis. JHEOR. 2021;8(1):106-115. doi:10.36469/jheor.2021.24564

Table S1. Regimen Risk

This supplementary material has been provided by the authors to give readers additional information about their work.

This is an open-access article distributed under the terms of the Creative Commons Attribution 4.0 International License (CCBY-4.0). View this license's legal deed at http://creativecommons.org/licenses/by/4.0 and legal code at http://creativecommons.org/licenses/by/4.0/legalcode for more information.

Regimen Risk Level	Regimens Included
High Risk	Cyclophosphamide, epirubicin every <20 days
	Docetaxel, cyclophosphamide (TC)
	Docetaxel, carboplatin, trastuzumab (TCH)
	Docetaxel, carboplatin, trastuzumab, pertuzumab
	Docetaxel, doxorubicin, cyclophosphamide (TAC)
	Doxorubicin, cyclophosphamide (AC) every <20 days
	Fluorouracil, epirubicin, cyclophosphamide
	Gemcitabine, carboplatin
	Methotrexate, cyclophosphamide, fluorouracil every <20 days
	Paclitaxel, carboplatin, trastuzumab
	Paclitaxel, carboplatin, trastuzumab, pertuzumab
	Paclitaxel every <20 days
	Trastuzumab, methotrexate, fluorouracil, cyclophosphamide every <20 days
Intermediate Risk	Carboplatin, etoposide
	Carboplatin, docetaxel
	Carboplatin, paclitaxel
	Cyclophosphamide, epirubicin every 21 days
	Cyclophosphamide, methotrexate
	Docetaxel
	Docetaxel, carboplatin, pertuzumab
	Docetaxel, trastuzumab
	Docetaxel, trastuzumab, pertuzumab
	Doxorubicin, cyclophosphamide (AC) for 1 cycle or every 21+ days
	Methotrexate, cyclophosphamide, fluorouracil every 21+ days
	Paclitaxel every 21+ days
	Paclitaxel, trastuzumab, pertuzumab
Other	Any regimen not defined as high- or intermediate-risk regimens
